# SI113, a SGK1 inhibitor, potentiates the effects of radiotherapy, modulates the response to oxidative stress and induces cytotoxic autophagy in human glioblastoma multiforme cells

**DOI:** 10.18632/oncotarget.7520

**Published:** 2016-02-19

**Authors:** Cristina Talarico, Vincenzo Dattilo, Lucia D'Antona, Agnese Barone, Nicola Amodio, Stefania Belviso, Francesca Musumeci, Claudia Abbruzzese, Cataldo Bianco, Francesco Trapasso, Silvia Schenone, Stefano Alcaro, Francesco Ortuso, Tullio Florio, Marco G. Paggi, Nicola Perrotti, Rosario Amato

**Affiliations:** ^1^ Department of “Scienze della Salute”, University “Magna Graecia” of Catanzaro, Catanzaro, Italy; ^2^ Department of “Medicina Sperimentale e Clinica”, University “Magna Graecia” of Catanzaro, Catanzaro, Italy; ^3^ Department of Farmacia, University of Genova, Genova, Italy; ^4^ Department of Medicina Interna e Specialità Mediche e Center of Excellence per la Ricerca Biomedica (CEBR), University of Genova, Genova, Italy; ^5^ Experimental Oncology, Regina Elena National Cancer Institute, IRCCS, Rome, Italy

**Keywords:** SGK1, SI113, radiotherapy, glioblastoma, oxidative stress

## Abstract

Glioblastoma multiforme (GBM) is the most aggressive CNS tumor and is characterized by a very high frequency of clinical relapse after therapy and thus by a dismal prognosis, which strongly compromises patients survival. We have recently identified the small molecule SI113, as a potent and selective inhibitor of SGK1, a serine/threonine protein kinase, that modulates several oncogenic signaling cascades. The SI113-dependent SGK1 inhibition induces cell death, blocks proliferation and perturbs cell cycle progression by modulating SGK1-related substrates. SI113 is also able to strongly and consistently block, *in vitro* and *in vivo*, growth and survival of human hepatocellular-carcinomas, either used as a single agent or in combination with ionizing radiations.

In the present paper we aim to study the effect of SI113 on human GBM cell lines with variable p53 expression. Cell viability, cell death, caspase activation and cell cycle progression were then analyzed by FACS and WB-based assays, after exposure to SI113, with or without oxidative stress and ionizing radiations. Moreover, autophagy and related reticulum stress response were evaluated.

We show here, that i) SGK1 is over-expressed in highly malignant gliomas and that the treatment with SI113 leads to ii) significant increase in caspase-mediated apoptotic cell death in GBM cell lines but not in normal fibroblasts; iii)enhancement of the effects of ionizing radiations; iv) modulation of the response to oxidative reticulum stress; v) induction of cytotoxic autophagy.

Evidence reported here underlines the therapeutic potential of SI113 in GBM, suggesting a new therapeutic strategy either alone or in combination with radiotherapy.

## INTRODUCTION

Malignant gliomas are the most frequent adult primary brain tumors and among these, glioblastoma multiforme (GBM) represent approximately 70% of glial tumors. Despite important advances in surgical techniques, chemotherapy, as well as conventional and stereotaxic radiotherapy approaches, for these patients the median of survival after diagnosis is only 12-15 months [1]. Since GBMs are highly heterogeneous, assessment of individual patient outcome, predicted by standard prognostic factors [2,3], can be improved by molecular approaches. Moreover, since a cure for malignant glioma remains elusive, it is important to develop new therapeutic strategies in order to inhibit tumor progression and restore therapeutic sensitiveness.

The serum- and glucocorticoid-regulated kinase 1 (SGK1) mediates signals of growth factor-dependent cell survival and proliferation [4]. SGK1 is a serine/threonine kinase that shares structural and functional similarities with the AKT family of kinases [5]. SGK1 function is tightly dependent on mTOR phosphorylation. Through the mTOR-dependent hydrophobic motif (H-motif) phosphorylation on serine 422 [6], the kinase acquires an open conformation for phosphorylation and full activation by 3-phosphoinositide-dependent kinase-1 (PDK1) [7]. SGK1 is regulated at different levels by insulin [8-10], IGF-1 [11], glucocorticoids [12] and IL-2 [13] and, in turn, modulates survival and proliferative signals in normal and cancer cells. Increased SGK1 expression has been found in several human tumors, including prostate [14] and non-small cell lung cancer [15]. Moreover, genome-wide analysis of gene expression in human hepatocellular carcinoma (HCC) cells demonstrates that SGK1 and its cognate kinase AKT1 are equally over-expressed when compared with normal human hepatocytes, suggesting that both kinases might have roles in hepatocellular dysregulation [16-18]. On the other hand, SGK1 knock-out models have been shown to be strongly resistant to chemical carcinogenesis [19]. It has recently been demonstrated that SGK1 regulates cell survival, proliferation and differentiation via phosphorylation of Mouse Double Minutes 2 (MDM2), which governs p53 ubiquitylation and proteosomal degradation [20]. SGK1 also affects mitotic stability in colon carcinoma cells by regulating the expression of RANBP1, the pivotal regulator of the GTPase RAN. SGK1 modulates RANBP1 abundance at the transcriptional level via SP1 activation and phosphorylation on Serine 59, thus affecting taxol sensitivity in these cells [21]. Taken together, all these lines of evidence point to SGK1 as a key element in the development and/or progression of human cancer.

Recently, we screened a family of dual SRC/ABL small molecule inhibitors, characterized by a substituted pyrazolo[3,4-d]pyrimidine scaffold, for their ability to inhibit SGK1 and AKT kinase activity, competing with ATP for their binding domain [22]. Among these molecules, SI113 resulted particularly effective in inhibiting SGK1 kinase activity, while being much less effective on AKT1, ABL and SRC activities [23]. Moreover, SI113 induces cell death and alters the growth rate in various malignant cell lines. Specifically, SI113 induces apoptosis in RKO colon carcinoma cells, either when used as a single agent or when synergizing with paclitaxel [23]. More recently, we presented impressive evidences on HCC cellular models *in vitro* as well as *in vivo* (xenografts in NOD/SCID mice), indicating that SI113 inhibits cancer cell proliferation, induces apoptosis and necrosis and potentiates the effects of radiotherapy, mimicking the effects of SGK1 knock-down. Based on the apparent lack of toxicity and the consistent effectiveness of SI113 in inhibiting tumor growth in mice models [24], we argued that this molecule is of potential value in the treatment of human HCC, either alone or in combination with radiotherapy [24]. In the present work, in a cohort of GBM patients, compared to non-tumor controls, we found that SGK1 expression correlated with high-grade glial tumors. Thus we expanded the analysis of SI113 efficacy in GBM cellular models and demonstrated that SI113 produces a dramatic decrease in cell viability by inducing apoptosis in GBM cell lines only, sparing normal mice fibroblasts. Consistent with our previous data, this drug enhances the effects of ionizing radiations in induction of cell death and distortion of cell cycle progression. In turn, SI113 synergizes with oxidative stress, the primary mechanism of the radio-dependent tumor killing, and modulates the autophagic response and the reticulum stress. Taken together, our data demonstrate the importance of SGK1 as molecular target in cancer therapy and the effectiveness of the SI113-dependent SGK1 inhibition also in GBM treatment, where this drug appears effective as a single agent and also in combination with radiotherapy.

## RESULTS

### SGK1 mRNA determination in tumor samples

**Figure 1:**
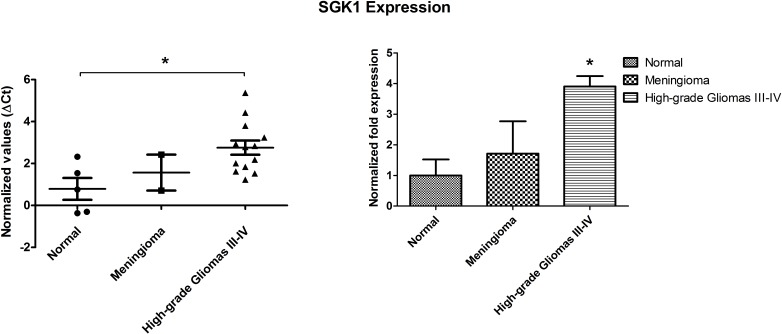
qPCR-based evaluation of SGK1 mRNA expression in brain tumors (glioblastoma multiforme and meningioma) compared with normal brain tissue Graphs (M+/− SE) represent the ΔCT distribution (left panel) and the fold increase values (right panel). Statistical significance (*P* = 0.01) has been calculated as detailed in the Methods section. *P ≤ 0.05; **P ≤ 0.01; ***P ≤ 0.001.

SGK1 expression was measured by means of real time PCR using SGK1-specific primers in tumor samples of meningioma, grade III malignant glioma and GBM, as well as in brain samples from non-tumor controls (Suppl. File 1). Hypoxanthine phosphoribosyltransferase mRNA was used as an internal check of quality and for normalization. GBM samples (*n*=13) showed a 3.9 fold increase in SGK1 mRNA expression compared to control samples (*n*=5) (*P*=0.01), whereas meningioma samples (*n*=2) were characterized by a level of SGK1 mRNA expression comparable with the one of normal brain tissue (Figure 1).

### GBM cell line characteristics

The protein expression p53 and p21was preserved in LI and ADF cells, whereas it was undetectable in A172 cells (Suppl. File 2).

### SI113 strongly reduces cell viability and induces caspase-dependent apoptosis in GBM cells, but not in normal murine fibroblasts

**Figure 2:**
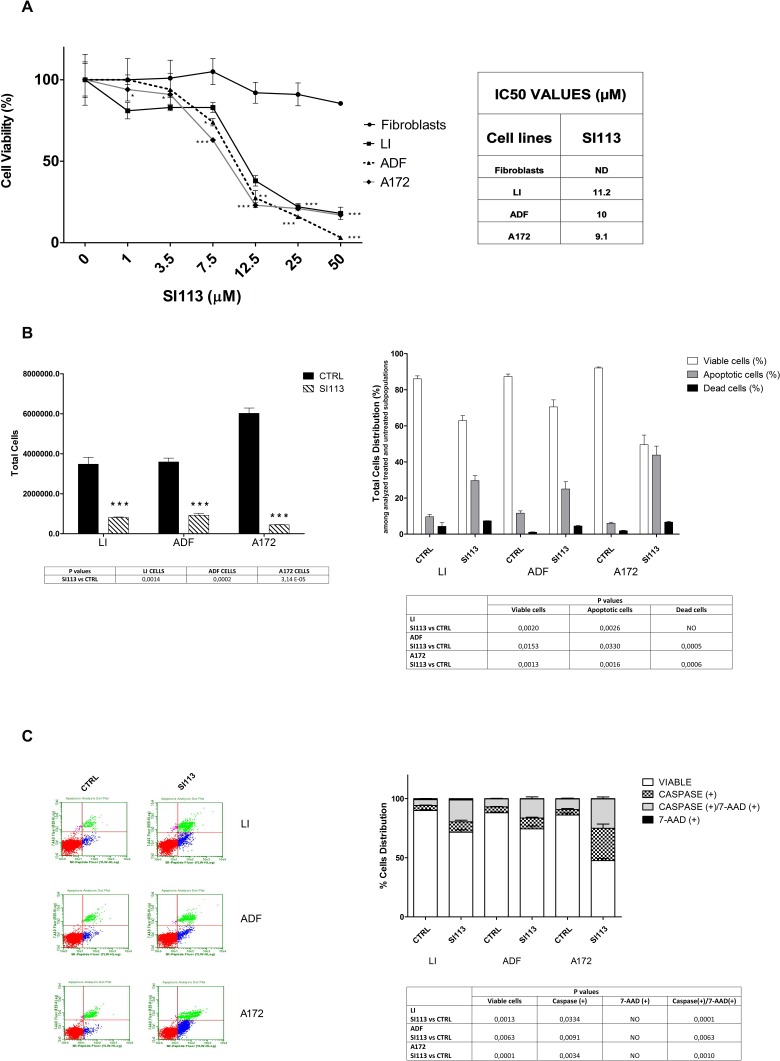
Cell growth inhibition and apoptosis induction by SI113 in LI, ADF and A172 human glioblastoma cell lines **A.** Cell viability analysis by The Countess™ automated cell counter in normal mouse stromal fibroblasts (MS5), LI, ADF and A172 cell lines 72 h after treatment with either SI113 at the indicated concentrations or vehicle alone. Results are reported as means of three independent experiments, each conducted in triplicate, and expressed as the percentage of viable control cells treated with DMSO alone (vehicle). The Table on the right reports the IC50 values for the GB cell lines. **B.** Left panel: The Bar Graphs represent the total number of cells (M+/−SE) treated with either SI113 (12.5 μM) for 72 h or vehicle alone, as indicated. Right panel: The Bar Graphs represent the distribution of viable/apoptotic/dead events among control and SI113 (12.5 μM for 72h) treated cells. Results represent the mean ± SE of six independent experiments for each cell line. **C.** Left panel: representative Guava caspase assay graphs of GBM cells lines treated with either SI113 (12.5 μM for 72 h) or vehicle alone. Right panel: Bar graphs represent the percentage of caspase and/or 7AAD positive cells after treatment with SI113 (12.5 μM for 72h). Statistical significances are reported in the Tables under the respective graphs. *P ≤ 0.05; **P ≤ 0.01; ***P ≤ 0.001.

Twenty-four hrs after plating, when cells were approximately 60% confluent (see Methods section) LI, ADF and A172 cells and normal fibroblasts (stromal mouse MS5 cells) were treated with SI113 and cell viability estimated 72h later by means of trypan blue Countess Assay. In all three GBM cell lines, SI113 yielded a significant and dose-dependent reduction in the number of viable cells (Figure 2, panel A left), replicating the results obtained in HCC cells [24]. Interestingly, SI113 had a very modest effect, if any, on cell viability in normal fibroblasts (stromal mouse MS5 cells), as predicted by the lack of toxicity observed when the drug was administered intra-peritoneally in murine models [24]. IC50 values for SI113 (0-50 μM, 72 hours), calculated for the 3 GBM cell lines, are listed in Figure 2 Panel A, right, and ranged from 9 to 11μM. IC50 value for normal fibroblasts was not determinable, since SI113 appeared to be virtually ineffective on these cells. In line with these data, from now on, SI113 has been employed at the concentration of 12.5 μM for 72 h, unless otherwise indicated. Figure 2, panel B, left, recapitulates in a dedicated experiment the effect of SI113 on GBM cell lines, under these experimental conditions.

Differential permeability of DNA-binding dyes and the forward scatter (FSC) properties allow the distinction of three different GBM cell populations: viable, apoptotic and necrotic/dead cells. In ADF and A172 cell lines, SI113 induced a consistent increase in apoptotic and necrotic populations (Figure 2, panel B, right), whereas in the LI cell line, only the apoptotic population resulted significantly increased under these conditions. SI113 significantly increased the number of non-viable cells in all three GBM cell lines (Suppl. File 3). A Guava Caspase assay was performed to verify and substantiate the amount and specificity of caspase-dependent apoptosis under SI113 treatment in these GBM cell lines (Figure 2, panel C, left). This particular assay allows the distinction between middle stage apoptosis [Caspase Reagent(+) and 7-aminoactinomycin D (7-AAD)(−)] and late stage apoptosis or death [Caspase Reagent(+) and 7-AAD(+)]. A significant increase in both middle and late stage apoptosis appeared evident in all the SI113-treated GBM cell lines, as indicated (Figure 2, panel C right top). The data indicate therefore that SI113 significantly activated the caspase-dependent apoptotic response pathways, as summarized in Figure 2, panel C, table, right, bottom).

### SI113 potentiates radiation-induced growth inhibition and cell cycle perturbation

**Figure 3:**
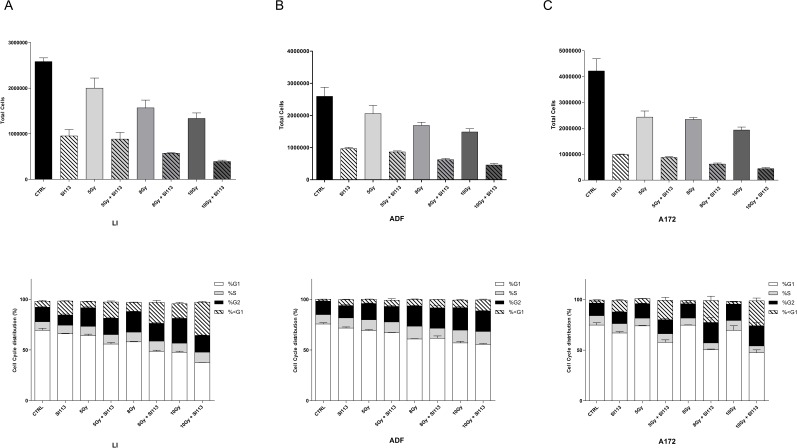
SI113 potentiates the effects of ionizing radiations in LI, ADF and A172 cells The bar graphs (Top Panels) represent the total number of cells treated with increasing dose of RT (5, 8, 10 Gy) in the presence or absence of SI113 (12.5μM) for 72 h. Results are expressed as the mean ± SE of six independent experiments for each cell line. The bar graphs (Bottom Panels) represent the cell cycle distribution of cells treated with increasing dose of RT (5, 8, 10 Gy) in the presence or absence of SI113 (12.5μM) for 72 h. Cells were analyzed by cytofluorimetry after staining with Guava Cell Cycle reagent. **A.** LI cell line, **B.** ADF cell line and **C.** A172 cell line. Statistical significances are reported in the tables under each graph. *P ≤ 0.05; **P ≤ 0.01; ***P ≤ 0.001.

The data described so far indicate a detrimental effect of SI113 on GBM growth and survival. We therefore explored the possibility that SI113-dependent inhibition of SGK1 might synergize with radiation therapy, as it was the case for HCC [24]. We evaluated the effect of SI113 on radiosensitivity of LI, ADF and A172 cells 24 h after plating. Cells were exposed to either no radiation (0 Gy) or radiation (5Gy, 8 Gy or 10 Gy), with or without the combined treatment with SI113, and assayed for cell viability by means of Guava ViaCount Assay. In each cell line analyzed, SI113, as a single agent, significantly reduced the number of viable cells, as expected. On the other hand, the sole radiation appeared effective only when used at 8 or 10 Gy. When both agents were used, the combination of SI113 and radiation reduced the number of viable cells significantly more than either agent alone (Figure 3, panel A, B and C Top and Suppl. File 4A) and Tab. 1 for statistics. In order to corroborate the effectiveness of this double treatment (SI113+RT), we carried out a FACS-based cell cycle analysis (Figure 3 Panel B bottom, Suppl. File 5B). In LI, ADF and A172 cells, a progressive increase in <G1, hypodiploic cells, together with a decrease in the percentage of G1 cells, was recorded when cells were exposed to increasing radiation doses and in the presence of SI113. In all three GBM cell lines, radiation as a single agent determined a significant and dose-dependent increase in the percentage of G2/M cells, and this effect was in part counteracted by SI113 in LI cells. (Fig. 3 Panel B bottom)

**Table 1:** Statistical significance of the differences observed in the viability and cell cycle distribution after treatment with ionizing radiation and /or SI113 as indicated

**A**						
**LIPARI**		**LIPARI**	**P values**			
	**P values**		**%G1**	**%S**	**%G2**	**%<G1**
**SI113 vs CTRL**	0,0005	**SI113 vs CTRL**	NO	NO	0,0212	0,0012
**5Gy vs CTRL**	NO	**5Gy vs CTRL**	NO	NO	0,0197	NO
**5Gy vs SI113**	0,0162	**5Gy vs SI113**	NO	NO	0,0032	7.36E-01
**5Gy+SI113 vs CTRL**	0,0005	**5Gy+SI113 vs CTRL**	0,0056	NO	NO	0,0019
**5Gy+SI113 vs 5Gy**	0,0135	**5Gy+SI113 vs 5Gy**	0,0175	NO	NO	0,0008
**5Gy+SI113 vs Si113**	NO	**5Gy+SI113 vs SI113**	0,0034	NO	0,0043	NO
**8Gy vs CTRL**	0,0058	**8Gy vs CTRL**	0,0047	0.0428	0,0007	0,0336
**8Gy vs SI113**	0,0498	**8Gy vs SI113**	0,0001	NO	0,0005	0,0003
**8Gy+SI113 vs CTRL**	1.72E-02	**8Gy+SI113 vs CTRL**	0,0007	0,0432	0,0453	0,0022
**8Gy+SI113 vs 8Gy**	0,0044	**8Gy+SI113 vs 8Gy**	0,0013	NO	0,0395	0,0037
**8Gy+SI113 vs Si113**	0,0562	**8Gy+SI113 vs SI113**	0,0001	NO	0,0052	0,0234
**10Gy vs CTRL**	0,0010	**10Gy vs CTRL**	0,0006	NO	0,0012	0,0370
**10Gy vs SI113**	NO	**10Gy vs SI113**	9,06E-05	NO	0,0006	NO
**10Gy+SI113 vs CTRL**	1.44E-02	**10Gy+5I113 vs CTRL**	8.70E-05	0,0478	0,0350	2.27E-02
**10Gy+SI113 vs 10Gy**	0,0017	**10Gy+SI113 vs 10Gy**	0,0006	NO	0,0022	0,0003
**10Gt+SI1113 vs SI113**	0,0178	**10Gy+SI113 vs SI113**	5,02E-07	NO	0,0034	2.01E-02
**B**						
**ADF**		**ADF**	**P values**			
	**P values**		**%G1**	**%S**	**%G2**	**%G1**
**SI113 vs CTRL**	0,0046	**SI113 vs CTRL**	NO	NO	NO	0,0014
**5Gy vs CTRL**	NO	**5Gy vs CTRL**	0,0171	NO	NO	0,0025
**5Gy vs SI113**	0,0134	**5Gy vs SI113**	NO	NO	0,0254	0,0377
**5Gy+SI113 vs CTRL**	0,0036	**5Gy+SI113 vs CTRL**	0,0030	NO	NO	0,0519
**5Gy+SI113 vs 5Gy**	0,0096	**5Gy+SI113 vs 5Gy**	NO	NO	NO	NO
**5Gy+SI113 vs Si113**	NO	**5Gy+SI113 vs SI113**	0,0177	NO	NO	NO
**8Gy vs CTRL**	0,0388	**8Gy vs CTRL**	0,0003	0,0096	0,0029	0,0002
**8Gy vs SI113**	0,0026	**8Gy vs SI113**	0,0007	0,0137	0,0017	NO
**8Gy+SI113 vs CTRL**	0,0023	**8Gy+SI113 vs CTRL**	0.0063	NO	0,0339	5,75E-06
**8Gy+SI113 vs 8Gy**	0,0005	**8Gy+SI113 vs 8Gy**	NO	0,0164	NO	0,0060
**8Gy+SI113 vs Si113**	0,0052	**8Gy+SI113 vs SI113**	0,0181	NO	0,0216	0,0066
**10Gy vs CTRL**	0,0209	**10Gy vs CTRL**	0,0007	NO	0,0025	0,0014
**10Gy vs SI113**	0,0095	**10Gy vs SI113**	0,0016	NO	0,0017	NO
**10Gy+SI113 vs CTRL**	0,0017	**10Gy+SI113 vs CTRL**	0,0002	0,0178	0,0024	0,0003
**10Gy+SI113 vs 10Gy**	0,0007	**10Gy+SI113 vs 10Gy**	NO	NO	NO	0,0312
**10Gy+SI113 vs SI113**	0,0015	**10Gy+SI113 vs SI113**	0,0003	0.0294	0,0014	0,0054
**C**						
**A-172**		**A-172**	**P values**			
	**P values**		%G1	**%S**	**%G2**	**%<G1**
**SI113 vs CTRL**	0,0159	**SI113 vs CTRL**	0,0435	NO	NO	0,0016
**5Gy vs CTRL**	NO	**5Gy vs CTRL**	NO	NO	NO	0,0098
**5Gy vs SI113**	0,0036	**5Gy vs SI113**	0,0060	NO	0,0174	0,0042
**5Gy+SI113 vs CTRL**	0,0140	**5Gy+SI113 vs CTRL**	0,0073	NO	NO	0,0055
**5Gy+SI113 vs 5Gy**	0,0028	**5Gy+SI113 vs 5Gy**	0,0026	NO	NO	0.0087
**5Gy+SI113 vs SI113**	NO	**5Gy+SI113 vs SI113**	0,0280	NO	NO	NO
**8Gy vs CTRL**	NO	**8Gy vs CTRL**	NO	NO	NO	NO
**8Gy vs SI113**	6,11E-05	**8Gy vs SI113**	0,0039	NO	NO	0,0016
**8Gy+SI113 vs CTRL**	0,0106	**8Gy+SI113 vs CTRL**	0,0005	0,0333	NO	0,0123
**8Gy+SI113 vs 8Gy**	3.41E-05	**8Gy+SI113 vs 8Gy**	5,54E-06	NO	NO	0,0124
**8Gy+SI113 vs SI113**	0,0012	**8Gy+SI113 vs SI113**	0,0003	0,0307	NO	NO
**10Gy vs CTRL**	0,0554	**10Gy vs CTRL**	NO	NO	NO	NO
**10Gy vs SI113**	0,0013	**10Gy vs SI113**	NO	NO	NO	0,0013
**10Gy+SI113 vs CTRL**	0,0088	**10Gy+SI113 vs CTRL**	0,0017	NO	NO	0,0013
**10Gy+SI113 vs 10Gy**	0,0002	**10Gy+5I113 vs 10Gy**	0,0145	NO	NO	0,0012
**10Gy+SI113 vs SI113**	0,0003	**10Gy+SI113 vs SI113**	0,0032	NO	NO	0,0101

### The SI113-dependent SGK1 inhibition potentiates the oxidative stress-mediated breakdown of cell viability

**Figure 4:**
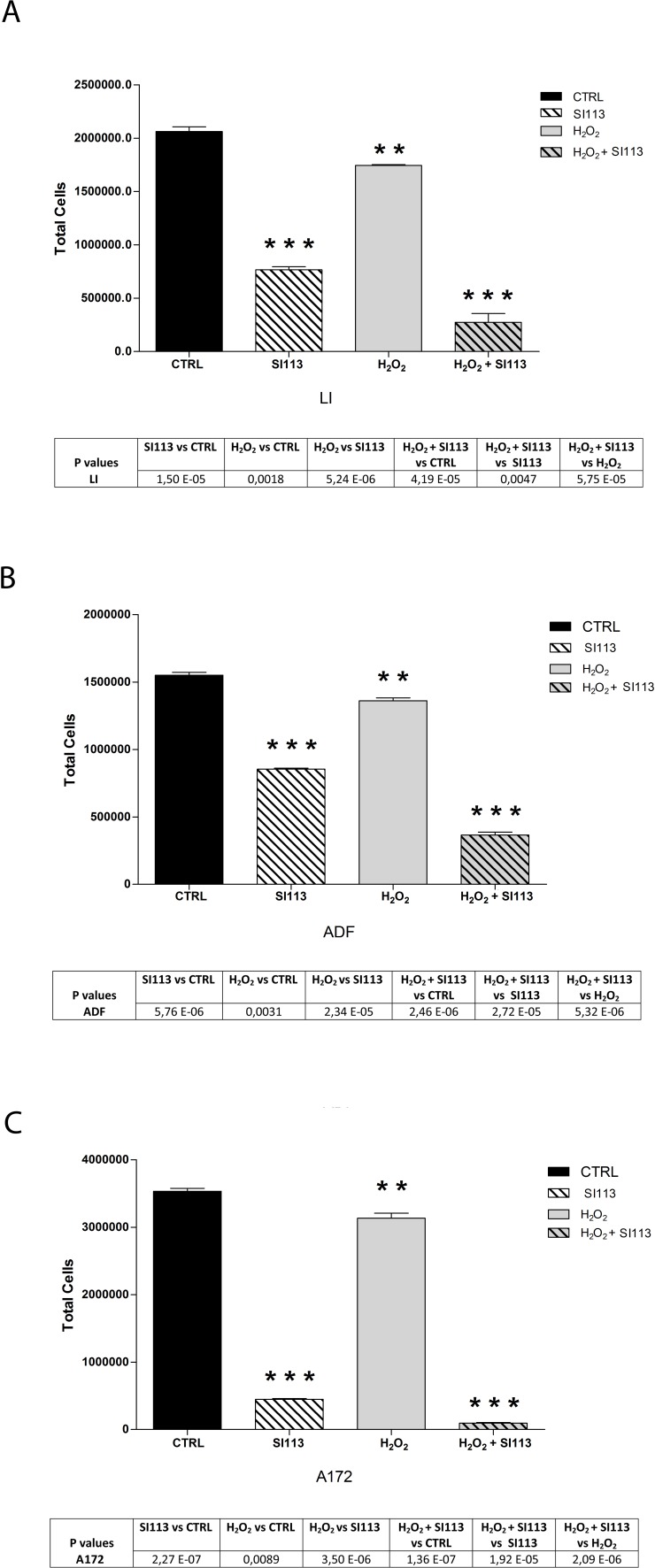
SI113 strongly potentiates the oxidative stress-mediated effects **A.** (LI), **B.** (ADF), **C.** (A172) The bar graphs represent the total number of cells treated or untreated with either H_2_O_2_ (250 μM) for 2 h or SI113 (12.5μM) for 72h or both agents together. Results are expressed as the mean ± SE of six independent experiments for each cell line. Statistical significances are reported in the Tables under each graph.*P ≤ 0.05; **P ≤ 0.01; ***P ≤ 0.001.

Oxidative stress is generally considered the primary mechanism of radiation-dependent cell killing, which is concordant with the dose-dependent increase of DNP-FITC positive cells upon exposure to increasing radiation doses (Suppl. File 6). SGK1, in turn, has been suggested as a protective factor that allows cell survival after oxidative stress [30, 31]. It is then conceivable that SI113 could enhance the effects of ionizing radiations on cell viability by direct SGK1 inhibition in GBM cells, as already demonstrated for HCC cells [24]. To test the ability of SI113 to influence the oxidative stress response, cell viability was estimated by means of Guava ViaCount Assay in GBM cells exposed to either no treatment or H_2_O_2_ (250μM for 2h), in the presence or absence of SI113. In each cell line, SI113, as single agents, significantly reduced the number of viable cells, as expected, whereas the effect of the sole H_2_O_2_ was barely significant, somehow comparable to the effect of the single radiation treatment. However, when the two treatments were co-administered, SI113 synergized with H_2_O_2_, reducing the number of viable cells significantly more than either agent alone (Figure 4, panel A, B and C and Suppl. File 7)

### Evidence supporting a protective role of SGK1 in the oxidative stress response

**Figure 5:**
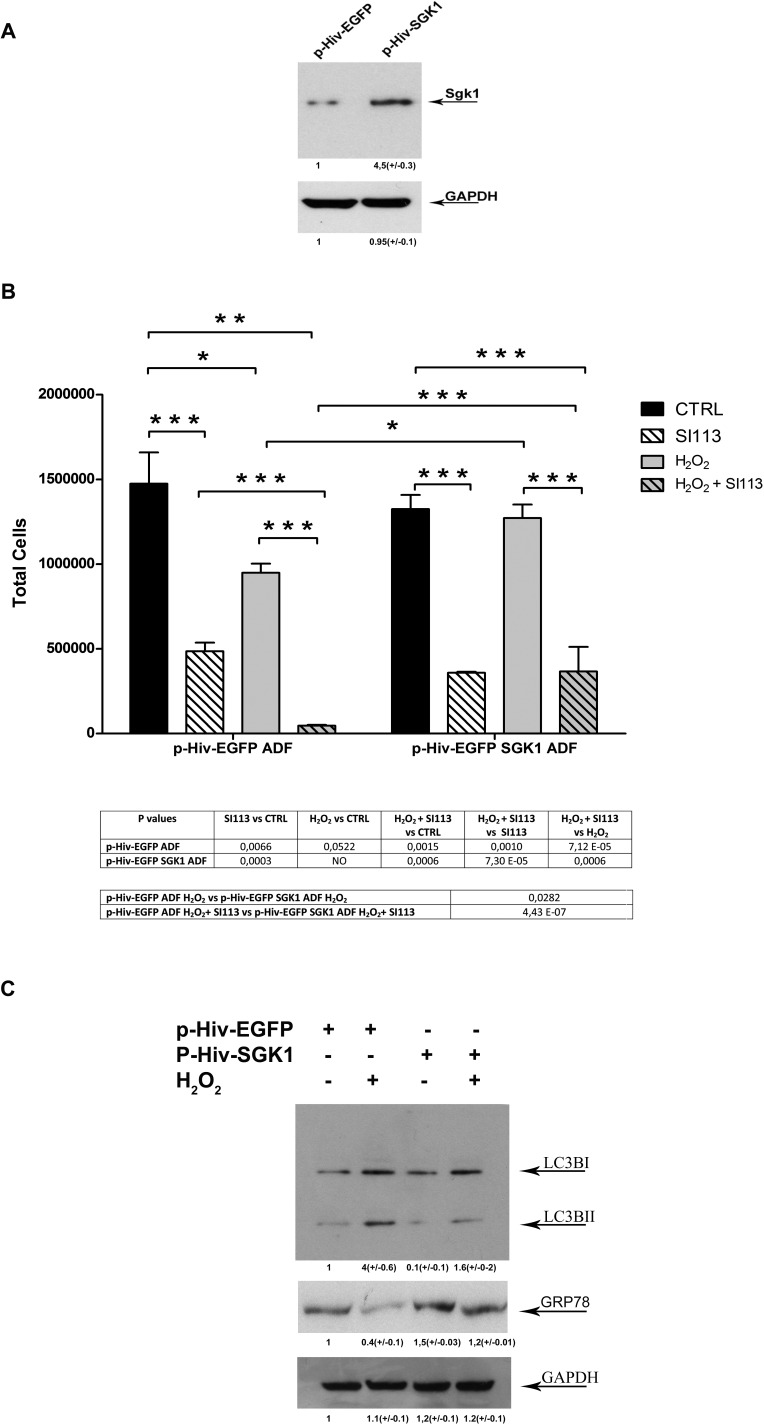
SGK1-dependent modulation of oxidative stress response **A.** Western blot analysis. SGK1 expression in p-HIV-EGFP ADF cells (left) and p-HIV-EGFP SGK1ADF cells (right). GAPDH was used as a loading control. **B.** The bar graphs represent the total number of p-HIV-EGFP ADF cells (left) or p-HIV-EGFP SGK1ADF cells (right), treated or untreated with either H_2_O_2_ (250 μM) for 2 h or SI113 (12.5μM) for 72 h. Results, obtained by means of Guava ViaCount assay, are expressed as the mean ± SE of six independent experiments for each cell line. Statistical significances are reported in the Tables under each graph.*P ≤ 0.05; **P ≤ 0.01; ***P ≤ 0.001. **C.** Western blot analysis. LC3B-I/LC3B-II conversion and GRP78 expression in p-HIV-EGFP ADF cells (left) and p-HIV-EGFP SGK1ADF cells (right), treated or untreated with H_2_O_2_ (250 μM) for 2h. GAPDH was used as a loading control.

To verify whether the level of SGK1 expression affected the cellular response to oxidative stress and the sensitivity to SI113, we used lentiviral vectors to produce stably transduced ADF cells expressing either wild type SGK1 (p-HIV- EGFP-SGK1ADF cells) or the related control vector p-HIV-EGFP (p-HIV-EGFP ADF cells). SGK1 over-expression was verified by Western blot (Figure 5, panel A). Considering the high proliferation rate typically observed in SGK1-overexpressing cell lines [21], different cell numbers were plated [p-HIV-EGFP-SGK1 (1.5 × 10^5^ cells)] and [p-HIV-EGFP (2.5 × 10^5^ cells)]. After 24 h, when the cells were approximately 60% confluent, SI113 or vehicle were added (Figure 5, panel B and Suppl. File 8). Cell proliferation and viability were estimated after 72 h by means of Guava viaCount reagents. Both vehicle-treated cultures had comparable cell numbers (p-HIV-EGFP cultures: 1474857 ± 319789 cells; p-HIV-EGFP-SGK1 cultures: 1324859± 145433 cells). In control p-HIV-EGFP cells (Figure 5, panel B, left), H_2_O_2_ significantly reduced the number of viable cells and, when H_2_O_2_ and SI113 were used together, the combined treatment reduced the number of viable cells much more than either agent alone. In p-HIV-EGFP-SGK1 cells (Figure 5, panel B, right), the effect of SI113 on cell viability was not affected by SGK1 over-expression. Interestingly SGK1 over-expressing cells appeared clearly protected from oxidative stress. In fact, H_2_O_2_ treatment did not reduce the number of viable cells in p-HIV-EGFP-SGK1 cells. Moreover, in these cells, the combined treatment with SI113 and H_2_O_2_ was much less effective than in control cells in reducing the number of viable cells, strongly resembling the effect of the sole SI113. Taken together, these data strongly support a protective role for SGK1 in oxidative stress-induced cell death.

It is generally believed that the activation of autophagy and the response to reticular stress can have a role in determining the cell fate upon oxidative stress [32]. LC3B-I/LC3B-II conversion is considered a marker of autophagosome degradation and cytotoxic autophagy [33], while GRP78 expression is usually activated under reticular stress as a survival and resolution factor. We used Western blot analysis to determine the expression levels of LC3B-I, LC3B-II and GRP78. The results revealed that H_2_O_2_ treatment increased the LC3B-I to LC3B-II conversion and, at the same time, down-regulated the expression of GRP78 (Figure 5, panel C, lanes 1 and 2 from left). Interestingly, SGK1 over-expression appeared to counteract the effects of H_2_O_2_ on both LC3B-I/LC3B-II conversion and GRP78 expression (Figure 5, panel C, lanes 3 and 4 from left), suggesting a protective role of SGK1 in cell death secondary to oxidative stress.

### The SI113-dependent SGK1 inhibition modulates autophagy response and stress response

The role of SI113 dependent inhibition of SGK1 on cell autophagic response was studied by staining with mono-dansyl-cadaverine (MDC) [34].

**Figure 6:**
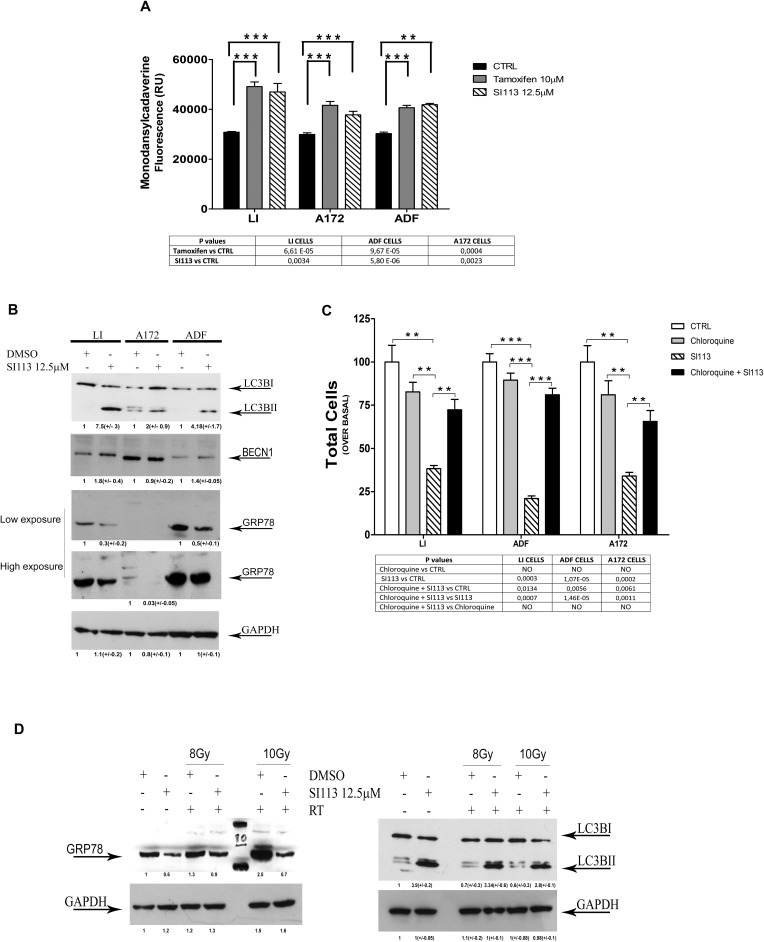
SI113 activates cytotoxic autophagy and modulates stress response to ionizing radiations **A.** Mono-dansyl-cadaverine (MDC)-labeled vesicles are induced by SI113; tamoxifen is used as a positive control. Bars graphs represent mean MDC incorporation as fold- of untreated control cells (Relative Units). GBM cells were incubated with either vehicle alone, SI113 or tamoxifen at the indicated doses for 72 h. Both treated and control cells were then incubated with MDC at 0.05 mM for 10 min at 37°C, as indicated in the Methods section, and analyzed by reader fluorescence detector. Data represent the mean ±SEM of at least six independent experiments. Statistical significances are reported in the Tables under the respective graphs. *P ≤ 0.05; **P ≤ 0.01; ***P ≤ 0.001. SI113. **B.** SI113 (12.5 μM) for 72 h enhances LC3B-I/LC3B-II conversion and BECN1 expression and decreases the expression of GRP78 in LI, A172 and ADF cell lines. GAPDH was used as a loading control. **C.** SI113 (12.5 μM) for 72 h reduces the number of viable LI, A172 and ADF cells and the effect is neutralized, in part, by pretreatment with chloroquine (CQ, 10 μM for 12 h). Bar graphs represent the number of untreated control cells, treated with either CQ alone, SI113 alone or both the agents, as indicated in Methods. Results are expressed as percent of untreated controls and represent the mean ± SE of three independent experiments for each cell line (The Countess™ automated cell counter after trypan blue stain). Statistical significances are reported in the tables at the bottom of graph. *P ≤ 0.05; **P ≤ 0.01; ***P ≤ 0.001. **D.** Left panel: SI113 (12.5 μM) for 72 h reduces the expression of GRP78 in ADF cells and counteracts the activation of GRP78 expression induced by ionizing radiations (8 Gy and 10 Gy). Right panel: SI113 (12.5 μM) for 72 h activates LC3B-I/LC3B-II, when used alone or in combination with ionizing radiations (8 Gy and 10 Gy). GAPDH was used as a loading control.

LI, A172 and ADF cell lines were plated at a comparable number; after 24h cells were treated with SI113 as usual. In all three GMB cell lines, SI113 treatment increased MDC fluorescence intensity, compared to the untreated controls, indicating that SGK1 inhibition caused an accumulation of autophagosome, possibly due to the block of the autophagic flux (Figure 6, panel A). The effect of SI113 was comparable with that of tamoxifen, widely regarded as an inducer of autophagy [35]. Moreover, in all GBM cells, SI113 induced LC3B-I to LC3B-II conversion and decreased the expression of GRP78 (Figure 6, Panel B; compare lanes 2,4 and 6 with lanes 1,3 and 5 from left), accordingly with the inhibition of SGK1 and the activation of autophagy. The expression of Beclin 1 (BECN1), the mammalian orthologue of yeast Atg6, that has a central role in autophagy [36] was clearly increased at least in LI and ADF cell lines. In order to discriminate between cytoprotective and cytotoxic autophagy, a vitality assay was carried out in GBM cells, pretreated with either chloroquine (10μM for 12h), an inhibitor of autophagy [37], or vehicle alone before the addition of SI113. Chloroquine treatment significantly decreased the SI113-dependent LC3B-I/to LC3B-II conversion, as expected (Suppl. File 9).

Moreover, the inhibition of autophagy by chloroquine antagonized the inhibitory effects of SI113 on cell viability (Figure 6, Panel C), thus suggesting that, at least in these GBM cells, SI113 dependent SGK1 inhibition activated cytotoxic autophagy, that can be inhibited to restore cell viability.

Finally we attempted to elucidate the molecular mechanisms underlying the combined effects of SI113 and radiation on cell viability. The expression of GRP78 is generally considered a cellular marker of radioresistance in tumors [38]. It has recently been suggested that GRP78 is induced by radiation in several cell lines, including malignant gliomas (D54 and GL261) [39-41]. In ADF cells, indeed, exposure to ionizing radiations increased the expression of the survival factor GRP78 in a dose-dependent manner (8 and 10 Gy), The radiation-dependent GRP78 induction was inhibited by SI113, thus suggesting a possible mechanisms through which this drug could enhance radiosentivity (Figure 6, Panel D left). The conversion of LC3B-I to LC3B-II is considered a marker of autophagy that, in our cells, appeared to be cytotoxic. Interestingly, in ADF cells, radiation exposure to 8 Gy and 10 Gy did not activate LC3B-I/LC3B-II conversion. On the other hand, treatment with SI113 clearly activated such conversion, in the absence or presence of ionizing radiations (Figure 6, Panel D right). Similar results were obtained in LI and A-172 cell lines (Suppl. File 10), suggesting another possible mechanism through which SI113 could contribute to potentiate the radiation-induced cell death.

### SI113 lipophilicity prediction

In order to consider the ability of compound SI113 to cross the blood-brain barrier, a preliminary pharmacokinetic evaluation was carried out *in silico* using the QikProp. We have recently used this tool also for selective monoaminoxidase B inhibitors based on the 3-acetyl-2-dichlorophenyl-5-aryl-2,3-dihydro-1,3,4-oxadiazole chemical scaffold, that, to be active in the Central Nervous System, must show good pharmacokinetic profiles [42]. In particular, the lipophilicity property, calculated by the QPlogPo/w descriptor, showed a value equal to 4.29, that is better than the best MAO-B inhibitors studied in our laboratory and compatible with a quite good blood-brain permeation.

## DISCUSSION

SGK1 plays a pivotal role in regulating the processes of neoplastic transformation and chemo/radio resistance [4, 13, 15, 19-21, 43-46]. SGK1 specific inhibitors have been tested in several neoplastic models, including colon and hepatocellular carcinoma [22-24, 47-49]. SGK1 is also considered a key element in the regulation of neuronal excitability [50], neuronal survival after stress exposure and axonal elongation [51, 52], whereas the role of SGK1 in the glial component is still elusive and yet to be determined. In several deposited Geoprofiles datasets, SGK1 expression correlates with grading in astrocytomas, including GBM, the highest grade. In the present paper, we show for the first time that an increased SGK1 mRNA expression was significantly enhanced in high-grade astrocytoma and GBM from well-staged patients, when compared with normal brain tissue. This evidence supports the idea that the level of SGK1 expression may represent a novel molecular marker in the phenotypic and functional characterization of GBM. For this reason, we decided to test the effect of a novel SGK1 inhibitor, SI113, [22, 24, 53] on GBM cell signaling and growth *in vitro*.

Our previous work suggested that SI113 was devoid of any acute toxic effect when administrated i.p. in experimental murine models [24]. Here we demonstrate that SI113 does not affect cell viability of normal mouse fibroblasts, over a wide range of concentration (1-50μM). In the same dose range, however SI113 dramatically and consistently reduced cell viability of GBM cells (IC50, ranging between 9.1 and 11.2μM). This selective behavior could reflect a different level of expression and/or activation of SGK1, together with a different metabolic condition between tumor and healthy cell models (e.g. chronical stress and oxidative bursting)[53]. Moreover, we provide evidence supporting the essential and limiting role of SGK1 in cancer cell survival. Infact, SI113-dependent SGK1 inhibition causes cell cycle delay and cell death, which interestingly recapitulates previously described effects of SGK1 silencing in several cancer cell lines [13, 20, 21, 23, 24]. In GBMs, the effects of SI113 on cell death, can be almost exclusively attributed apoptosis, whereas in other cellular systems either mitotic death or necrotic phenomena were also observed [21, 24].

Conventional or stereotactic radiotherapy represents the gold standard as a first line treatment for the majority of high grade glial neoplasms, which are frequently unresectable at diagnosis [1, 54]. SGK1 is emerging as a key element in the control of tissue specific radio-sensitivity [46, 47, 49, 55]. In the present paper, we show that SI113-dependent SGK1 inhibition enhanced the effects of radiation therapy. Notably, ionizing radiations as a single agent, determined a mild cytotoxic response, mainly detected at the highest doses. When SI113 was administered before radiation therapy, at each dose the effect of the combined therapy was more remarkable than the effect of either agent alone. Cell cycle distribution revealed the progressive increase in hypodiploid cells (<G1), as well as the progressive decrease in G1 cells, with either agent and with the combined therapy in all the three GBM cell lines. A significant increase in the percentage of G2/M cells was observed when radiation was used as a single agent, consistent with previously published observations [56]. Notably, only in LI cells, SI113 determined a significant decrease in the percentage of G2/M cells, an effect that we previously observed in other cells lines [21, 24]. Interestingly, in these cells, SI113 appeared to counteract the radiation-induced increase in the percentage of G2/M cells. In order to provide a better understanding of the molecular mechanisms underlying the combined effect of SI113 and ionizing radiation, we decided to focus on the response to oxidative stress. Free radicals production and the consequent oxidative stress, is considered the main mechanism for ionizing radiations-dependent cytotoxicity, especially in the sub-lethal response [57]. Various evidence in noncancerous mouse mammary cell lines, as well as in the transgenic model of amyotrophic lateral sclerosis (ALS), suggest a role for SGK1 in mediating cell survival in response to oxidative stress [58, 59]. However, few studies explore this subject in human cancer cellular models, and the results appear somehow patchy [60]. Here we show that SI113 synergizes with H_2_O_2_ in reducing cell viability of the assayed GBM cell lines, thus suggesting that the SI113-dependent SGK1 inhibition, similarly to what observed upon ionizing radiation, potentiates the apoptotic response to free radicals. In order to confirm the protective role of SGK1 in GBM cells response to oxidative stress, we engineered the ADF cell line to stably over-express SGK1 and demonstrated that these cells are indeed more resistant to oxidative stress. Since SGK1 inhibition increased, whereas SGK1 over-expression decreased the sensitivity to oxidative stress, we conclude that SGK1 expression appears essential and rate-limiting in the modulation of the oxidative response in GBM tumor models.

This finding is complementary with our previous evidences that show a complete loss of activity of SI113 in HCC cells in which SGK1 expression was stably silenced by specific shRNA [24]. Cancer cells expressing high levels of proteins that promote survival after oxidative stress may be more resistant to radiation by means of different integrated mechanisms such as autophagy and reticulum stress response [38, 61-63]. Endoplasmic reticulum (ER) provides the oxidative environment that favors the formation of disulfide bonds, with generation of reactive oxygen species (ROS). Under these circumstances, the activation of the unfolded protein response (UPR) represents an adaptive mechanism to preserve cell survival [32]. The ER chaperone GRP78 functions as a potent anti-apoptotic factor and is usually activated under reticular stress as a survival and resolution factor that may confer resistance to radio- and chemotherapy [64]. We have presented evidence that H_2_O_2_ induced autophagy, as detected by the increased LC3B-I/LC3B-II conversion and, concomitantly, down-regulated the expression of GRP78, the main protein that promotes survival in response to reticulum stress [63] suggesting, in this model, a critical role of autophagy in cytotoxicity induced by H_2_O_2_. Interestingly, both these H_2_O_2_-dependent effects on LC3B-I/LC3B-II conversion and GRP78 expression were inhibited by SGK1 over-expression, suggesting its protective role in cell death response to oxidative stress. These data confirm previously published results demonstrating that mTOR downstream signals disclose an important role in modulating the autophagic response [29, 62, 65]. Having established the critical role of SGK1 in modulating autophagic response under oxidative stress, we wondered whether SGK1 inhibition could, in turn, modulate the levels of basal autophagy in GBM cell lines. We analyzed the regulation of autophagy after staining with mono-dansyl-cadaverine (MDC), a probe for detection of late autophagic vacuoles that is incorporated into multilamellar bodies by both an ion trapping mechanism and interaction with membrane lipids. We showed that SI113-dependent SGK1 inhibition activated autophagy as detected by enhancement of MDC fluorescence. Confirmative results were obtained by the analysis of LC3B-I/LC3B-II conversion, Beclin 1 expression and down-regulation of the expression of GRP78 in all three GBM cell lines.

Taken together, all these data support the essential and rate-limiting role of SGK1 in modulating autophagic and survival response to oxidative and reticulum stress. Interestingly, chloroquine, an inhibitor of autophagy [66], inhibits the effects of SI113 on LC3B-I/LC3B-II conversion and inhibits the SI113 effects on cell viability, suggesting that SI113 can induce a cytotoxic form of autophagy.

Moreover, a preliminary pharmacokinetic profile, carried out by the prediction of the lipophilicity of SI113, indicates a good probability of blood-brain permeation also if administered in a different way.

We finally analyzed the combined effects of ionizing radiation and SI113 on LC3B-I/LC3B-II conversion and on GRP78 expression as a measure of autophagy. Surprisingly, we found that ionizing radiation induced a dose-dependent increase in the expression levels of GRP78, whereas had no effect on the activation of LC3B-I/LC3B-II conversion. This may explain the low level of cell death recorded under treatment with ionizing radiations alone. In fact, GRP78 has been recently described as a radiation-induced survival factor that is related to the development of radioresistance [38]. On the other hand, failure in the activation of cytotoxic autophagy, has been proposed as a mechanism of partial or total chemo- and radio-resistance [67, 68]. We demonstrate that the SI113-dependent inhibition of SGK1 was able to down-regulate GRP78 expression and induce the LC3B-I/II conversion even in the presence of radiotherapy, thus overcoming survival mechanisms activated by the cell in response to radiation.

In conclusion, we present evidence supporting an important role for SGK1 in cell survival response to radiation and oxidative stress. SI113-dependent SGK1 inhibition counteracts the activation of these survival mechanisms and enhances the cell death in response to radiation and oxidative stress. Among the most relevant survival mechanisms, we discuss the activation of the expression of GRP78, that is clearly inhibited by SI113. Besides that, SI113 dependent inhibition of SGK1 also enhances cytotoxic autophagy that leads the cells exposed to radiation to an irreversible death fate. In a previous paper [24], we used SI113 in a murine xenograft model of liver cancer demonstrating its effectiveness, in the absence of apparent toxic effects. We now propose the SI113 dependent inhibition of SGK1 as a powerful therapeutic tool to overcome radio-resistance and enhance therapeutic effectiveness to the standard GBM radiotherapy.

## MATERIALS AND METHODS

### GBM cell lines

Human GBM cell lines A172 [25], ADF [26] LI [27] were kindly provided by one of us (M.G.P.). A172 cell lines were grown in Dulbecco's modified Eagle's medium (DMEM), while ADF and LI cell lines were grown in RPMI and MS5, a murine stromal cell line, in Alpha-MEM. All culture media were from Life Technologies, Inc., Grand Island, NY, and were supplemented with 10% fetal bovine serum and 1% penicillin-streptomycin solution (GIBCO). Cells were cultured at 37°C in a humidified atmosphere of 5%CO_2_ and 95% air.

### Recombinant DNAs

Preparation of p-HIV-EGFP expression vector for SGK1 expression, p-HIV-EGFP-SGK1

The procedure was performed according to Talarico et al. [24]

### Human brain tumor samples

Samples, collected by the Neurosurgery Unit of the IRCCS-AOU, San Martino IST (Genova, Italy) from 2005 and 2007, were supplied by one of us (T.F.). After surgery, tumor specimens were immediately frozen at −80°C till the processing for mRNA extraction. Non-tumor brain samples derive from the Brain Bank at Case Western Reserve University (Cleveland, OH) and are a kind gift of Prof. Claudio Russo (University of Molise, Italy). Patients' and samples' characteristics are detailed in the Table in the Suppl. File 1.

### Quantitative real-time PCR

RNA extraction was performed with RNeasy^®^Mini Kit (Qiagen, Valencia, CA, USA), following manufacturer's instructions. Tissues were disrupted with the aid of a homogenizer (IKA^®^-WERKE T8.01). Total RNA quality and quantity was evaluated by 260/280 nm reading ratio using the Multiskan Go spectrophotometer (Thermo Scientific, Madison, WI, USA) and by agarose gel electrophoresis. One μg of total RNA was subjected to reverse-transcription using the High Capacity RNA-to-cDNA Kit (Applied Biosystems, Foster City, CA, USA), following manufacturer's instructions. One μl (50ng) of cDNA was amplified by real-time PCR with SYBR^TM^Green master mix 2X (Promega, Madison, WI, USA) and 10 pmol of primers in a total volume of 20 μl. The specific primers for*SGK-1*were as follows:

52-GGCACCCTCACTTACTCCAG-3′(forward primer)
5′-GGCAATCTTCTGAATAAAGTCGTT-3′ (reverse primer).

Specific oligonucleotides used for hypoxanthine phosphoribosyl transferase (normalization control) amplification were reported in [28]. Accurate normalization of real-time quantitative RT-PCR data was carried out by geometric averaging of multiple internal control genes (Genome Biol 3, 0034). Reactions were performed in triplicate for each sample and were carried out in a BioRadiQ^TM^5 apparatus with the following conditions: initial denaturation step at 95°C for 10 min, followed by 40 cycles of 10 s at 95°C and 1 min at 57°C. Specificity of PCR products was checked by melting curve analysis.

### SI113 treatment

SI113 was developed as previously reported [22]. The drug was diluted in dimethylsulfoxide (DMSO) at a 10mM initial concentration and stored at −20°C.

### Radiation therapy

Cells were plated in 100-mm Ø tissue culture dishes, allowed to attach for 24 h, and treated with different doses of radiation (5, 8 and 10 Gy) at room temperature (1.8 Gy/min, 98 cm Source Surface Distance (SSD) by using a 6 MV photon linear accelerator (CLINAC 600 Varian) [29].

### Viability assay

For IC50 evaluation, cell proliferation and viability assay was performed via The Countess™ automated cell counter (Catalog no. C10227, Invitrogen) using trypan blue stain. The other experiments were carried out by means of Guava ViaCount Assay. To characterize SI113-dependent autophagy, 2 × 10^5^ cells were plated in 6-well tissue culture plates, pretreated with either chloroquine diphosphate 10μM for 12h (C6628 Sigma-Aldrich) or vehicle, followed by either SI113 12.5μM for 72h or vehicle.

### Guava ViaCount assay

An amount of 1.5 × 10^4^ cells was plated in 60-mm Ø tissue culture dishes, allowed to attach for 24h; then cells were incubated in the absence or presence of 12.5μM SI113 for 72h. To evaluate the effects induced by hydrogen peroxide (H_2_O_2_), 3 × 10^5^ cells were seeded in six-well plates, treated with SI113 as described, and finally treated with H_2_O_2_ (250μM) for 2 h. For radiation therapy, cells were treated with different doses of radiation (5, 8 and 10 Gy) at room temperature, in the presence or absence of SI113 (12.5 μM for 72h). Finally, each sample was prepared according with manufacturer's instructions (4000-0040, Millipore, Guava ViaCount Reagent for Flow Cytometry) for staining by mixing with ViaCount Reagent at a 20-fold dilution. After 5 min incubation at room temperature in the dark, samples were ready to be acquired on a Guava System.

### Caspase assay

To allow direct determination of the percent of live, apoptotic, dead and necrotic populations, distinguished by the presence or absence of activated caspases and/or an intact plasma membrane, 2 × 10^4^ GBM cells/ml, untreated or treated with SI113 12.5μM for 72h, were used for the Guava Caspase Assay according with the manufacturer's instructions (Millipore, 4500-0500). Four populations of cells were thus distinguished in this assay: Lower-left quadrant: viable cells [Caspase Reagent(−) and 7-AAD(−)]; Lower-right quadrant: cells in the middle stages of apoptosis [Caspase Reagent(+) and 7-AAD(−)]; Upper-right quadrant: cells in the late stages of apoptotic or dead [Caspase Reagent(+) and 7-AAD(+)]; Upper-left quadrant: necrotic cells [Caspase Reagent(−) and <7-AAD(+)].

### Cell cycle analysis

Guava Cell Cycle Reagent (Millipore, 4500-0220) staining was used to evaluate cell cycle effects of SI113 alone or in combination with radiation therapy at 8 and 10 Gy.

LI, ADF and A172 cells were treated for 72h with SI113, according with manufacturer's instructions and acquired on the Guava instrument. The DNA bar graph shows the result for the percentage of cells in G0/G1, S, G2/M under % Total.

### Oxidative stress assay

ADF Cells were plated in 25 cm^2^ tissue culture flasks, allowed to attach for 24 h and treated with three different doses of radiation (5,8 and 10 Gy) at room temperature (1.8 Gy/min, 98 cm Source Surface Distance (SSD) by using a 6 MV photon linear accelerator (CLINAC 600 Varian). After 72h, cells were processed using the Flow Cellect Oxidative Stress Characterization Kit. Flow Cellect (FCCH025111, Millipore)

Cells were analyzed on Guava EasyCyte Plus flow cytometer. The median fluorescence intensity (MFI) was calculated: an increase in oxidative stress is detected as a right shift in MFI from untreated sample to the treated sample.

### Western blot analysis

Cells treated as described, were processed as previously indicated [21] and probed with anti-MAP- LC3 (H-50) rabbit polyclonal antibody (sc-28226, Santa Cruz Biotechnology, Inc. Santa Cruz, CA), anti-p53 (DO 1) mouse monoclonal antibody (sc-126, Santa Cruz Biotechnology, Inc. Santa Cruz, CA), anti-p21 (C-19) rabbit polyclonal antibody (sc-397, Santa Cruz Biotechnology, Inc. Santa Cruz, CA), anti-SGK1 rabbit polyclonal antibody (#07-315, MERCK MILLIPORE), anti-BIP(GRP-78) (C50B12) rabbit polyclonal antibody (#3177, Cell Signaling Technology, Inc.) anti-GAPDH (sc-25778, Santa Cruz Biotechnology, Inc. Santa Cruz, CA), Anti BECN1 (sc-48381, Santa Cruz Biotechnology, Inc. Santa Cruz, CA)

### Autophagy/Cytotoxicity assay

Autophagy was induced by incubating GBM cells for 72h in the absence or presence of either tamoxifen (10μM) or SI113 (12.5μM). Cells were then trypsinized and an equal number of cells (5.0×10^4^ cells/well) were seeded in 96-well plates and incubated in PBS containing mono-dansyl-cadaverine (MDC) (0.05 mM) at 37°C for 10 min, according with manufacturer's instructions (Autophagy staining Kit, 600140, Cayman Chem. Co.). Fluorescence was quantified using a Fluoro count plate reader (excitation wavelength 380 nm, emission filter 525 nm). The MDC incorporated was expressed as specific activity (relative units).

### Predicted lipophilicity

SI113 was submitted to a preliminary lipophilic evaluation using the in silico method (Qikprop, in: Schrödinger Suite, Schrödinger LLC., New York, NY, USA.). Among several ADME descriptors the QPlogPo/w, corresponding to the predicted octanol/water partition coefficient, was considered as the most appropriate to predict the blood-brain permeation.

### Statistical analysis

All tests were done in triplicate and experiments performed at least three times. The results are expressed as a mean ± Standard Error (SE). Differences between groups were analyzed using the Student's two-tailed *t* test (GraphPadPrism v5 software, www.graphpad.com). Asterisks denote statistical significance as indicated in the legends.

## SUPPLEMENTARY MATERIAL FIGURES


