# Supplementary material for: SI113, a SGK1 inhibitor, potentiates the effects of radiotherapy, modulates the response to oxidative stress and induces cytotoxic autophagy in human glioblastoma multiforme cells
Source: Oncotarget. 2016 Feb 19;7(13):15868–84. doi: 10.18632/oncotarget.7520 (PMC4941283; doi:10.18632/oncotarget.7520)
Supplement: Supplementary file 1 [file oncotarget-07-15868-s001.pdf]

SI113, a SGK1 inhibitor, potentiates the effects of radiotherapy, modulates the response to oxidative stress and induces cytotoxic autophagy in human glioblastoma multiforme cells

Supplementary Material

							Immunoistochemistry				
		**BRAIN TUMORS**	**Sex**	**Age**	**Subtype**	**Anatomic location**	**MIB1**	**p53**	**GFAP**	**ER/PR**	**Notes**
	65G/07	GBM IV	F	68	Secondary	rolandic, Dx					
	62G/07	GBM IV	M	57	Primary						
	9G/05	GBM IV	M	65	Primary		10%			ER-/PR-	
	15G/05	GBM IV	F	49	Primary	rolandic	30%		pos		
	G2	GBM IV	M	65	Sercondary						
	39G/06	GBM IV	M	76	Primary	temporal					
	28G/06	GBM IV	M	65	Primary	temporo-parietal, Dx	>50%	20%	pos		
	10G/05	GBM IV and anaplastic Astrocitoma (Mixed)	M	74	Primary		20%				
	70G/07	Glioblastoma (GBM) with oligodendroglioma component (GBMO)	F	60	Primary	frontal, Sx	40%				
	7G/05	OLIGO/ASTROCYTOMA WHO II / III (Mixed)	M	29	Primary		5,50%				
	G1	OLIGODENDROGLIOMA WHO II	F	42	Primary	temporal					infiltrative
	29G/06	OLIGODASTROCITOMA WHO III	M	66	Relapse		<10%	<5%			previous GBM IV
		**NON-TUMORAL BRAIN TISSUES**									
**N1**	A83-123	PROGRESSIVE SUPRANUCLEAR PALSY (PSP)	M	76		FRONTAL CORTEX					
**N2**	A86-076	M. PARKINSON	M	45		FRONTAL CORTEX					
**N3**	A96-311	CORTICOBASAL GANGLIONIC DEGENERATION	F	65		FRONTAL CORTEX					
**N5**	A96-229	AD MILD, ATHEROSCLEROSIS OF THE ARTERIES OF WILLIS CIRCLE	F	60		FRONTAL CORTEX					
**N6**	A97-53P	AD, MODERATE	M	71		FRONTAL CORTEX					

Supplementary Figure 1 Table. Patients’ and samples’ characteristics.

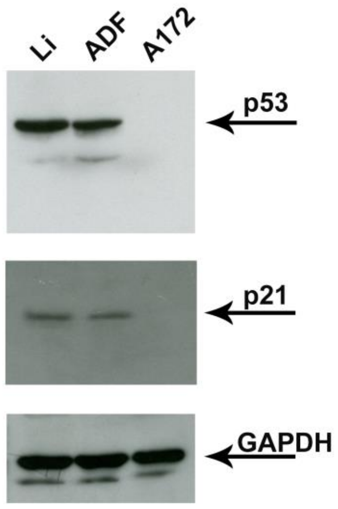

Supplementary Figure 2 p53 and p21 expression in LI, ADF and A172 GBM cell lines. GAPDH was used as a loading control.

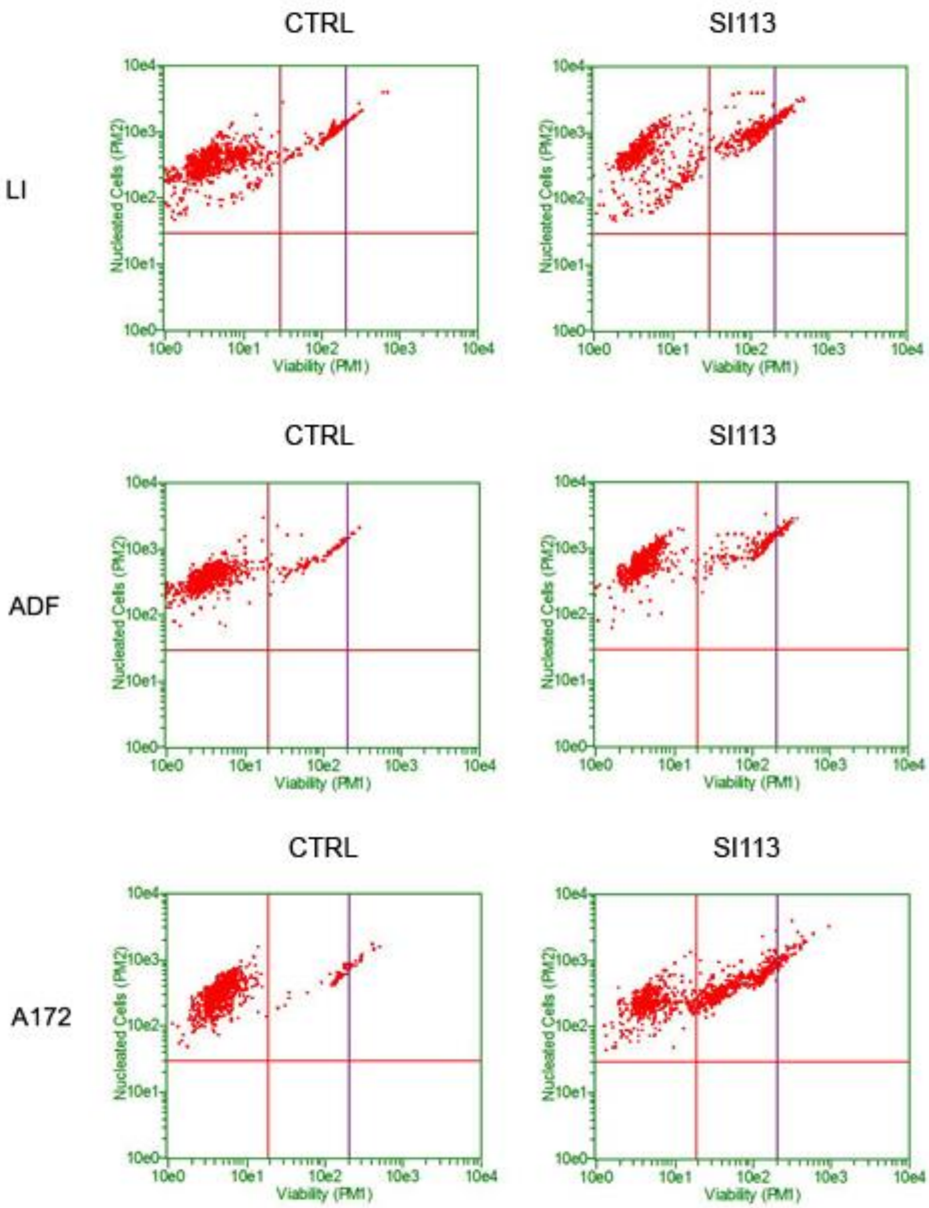

Supplementary Figure 3 histograms analyzed by Guava ViaCount flow cytometer**,** related to cells treated with SI113 (12.5 μM) for 72 or untreated. Distribution of viable/apoptotic/dead events among control and SI113 (12.5mM-72h) treated cells is gated in the figure.

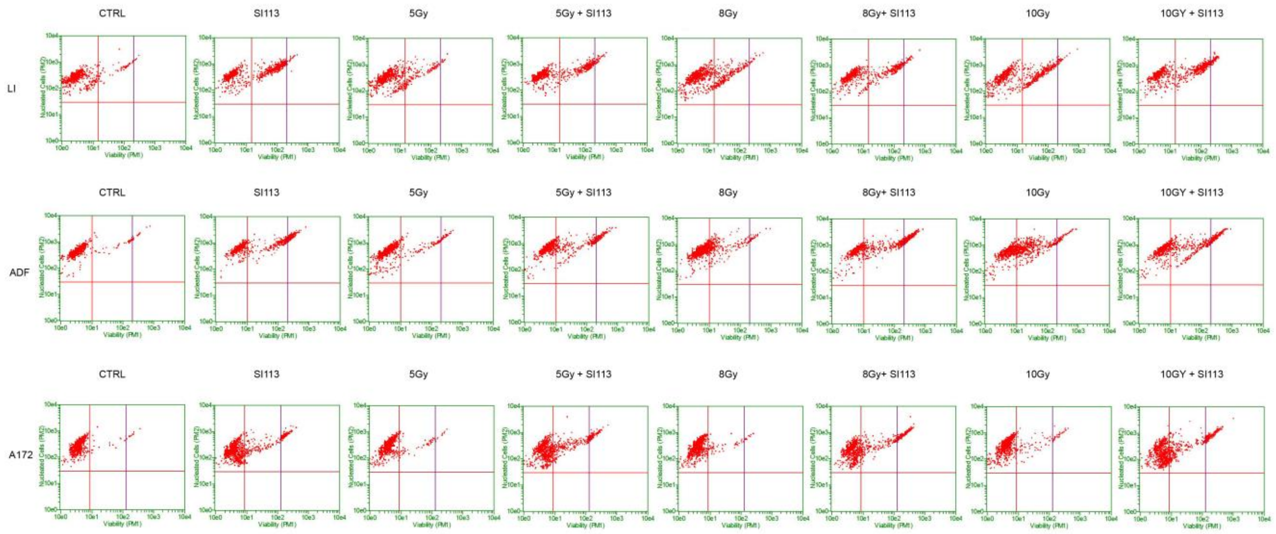

Supplementary Figure 4 Representative histograms of cells (LI, ADF and A172) treated with both increasing dose of RT (5, 8, 10 Gy) and SI113 (12.5 μM) for 72 h and analyzed by Guava ViaCount flow cytometer

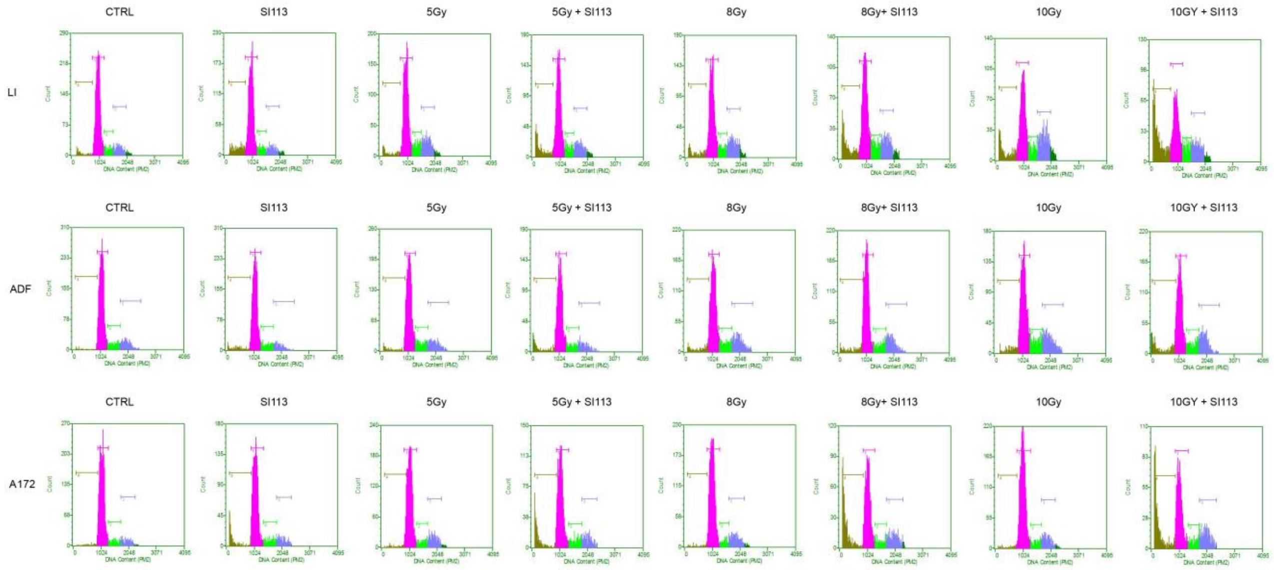

Supplementary Figure 5 Histograms of cell cycle analysis for LI, ADF and A172 GBM cell line treated with both increasing dose of RT (5, 8, 10 Gy) and SI113 (12.5 μM) for 72 h and analyzed by Guava CellCycle flow cytometer

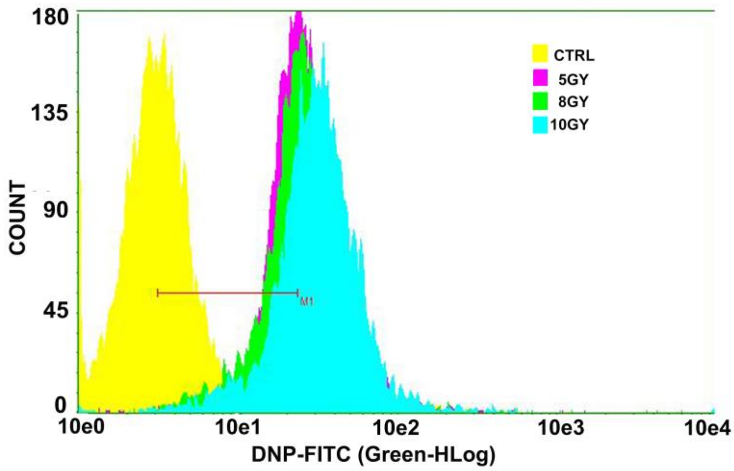

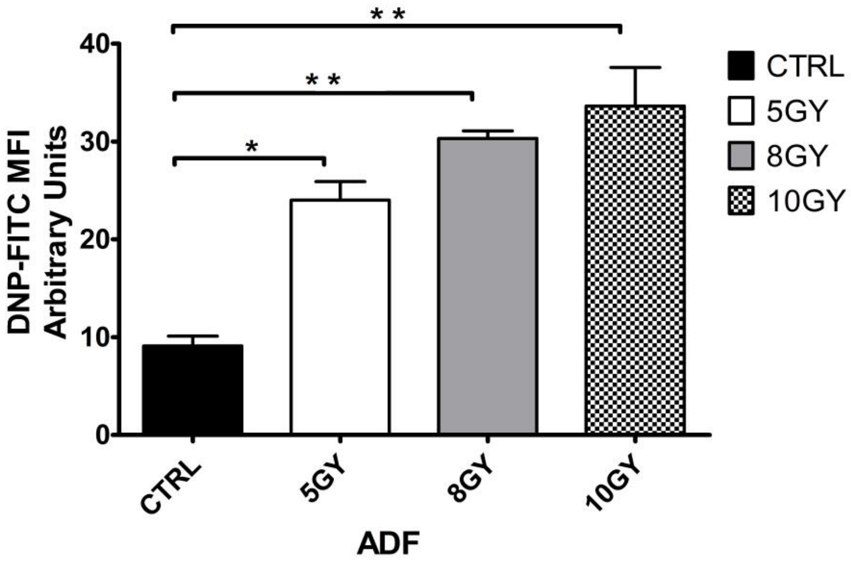

Supplementary Figure 6 The histograms represent the median fluorescence intensity (MFI) of treated and untreated ADF cells for 72 h with respectively three different doses of radiation 5, 8 and 10 Gy. After 72h cells were processed by means of the FlowCellect Oxidative Stress Characterization Kit and analyzed by Guava EasyCyte Plus flow cytometer. Values for treated and untreated cells in the different conditions were compared by One-way analysis of variance (ANOVA) P values= 0,0003. Results represent mean ± S.D. of three independent experiments. Statistical significance was calculated by Bonferroni's Multiple Comparison Test : ADF cells. 5 Gy vs CTRL cells *P* ≤0,005, 8 Gy vs CTRL cells *P*≤0.001, 10 Gy vs CTRL cells *P*≤ 0.0001.

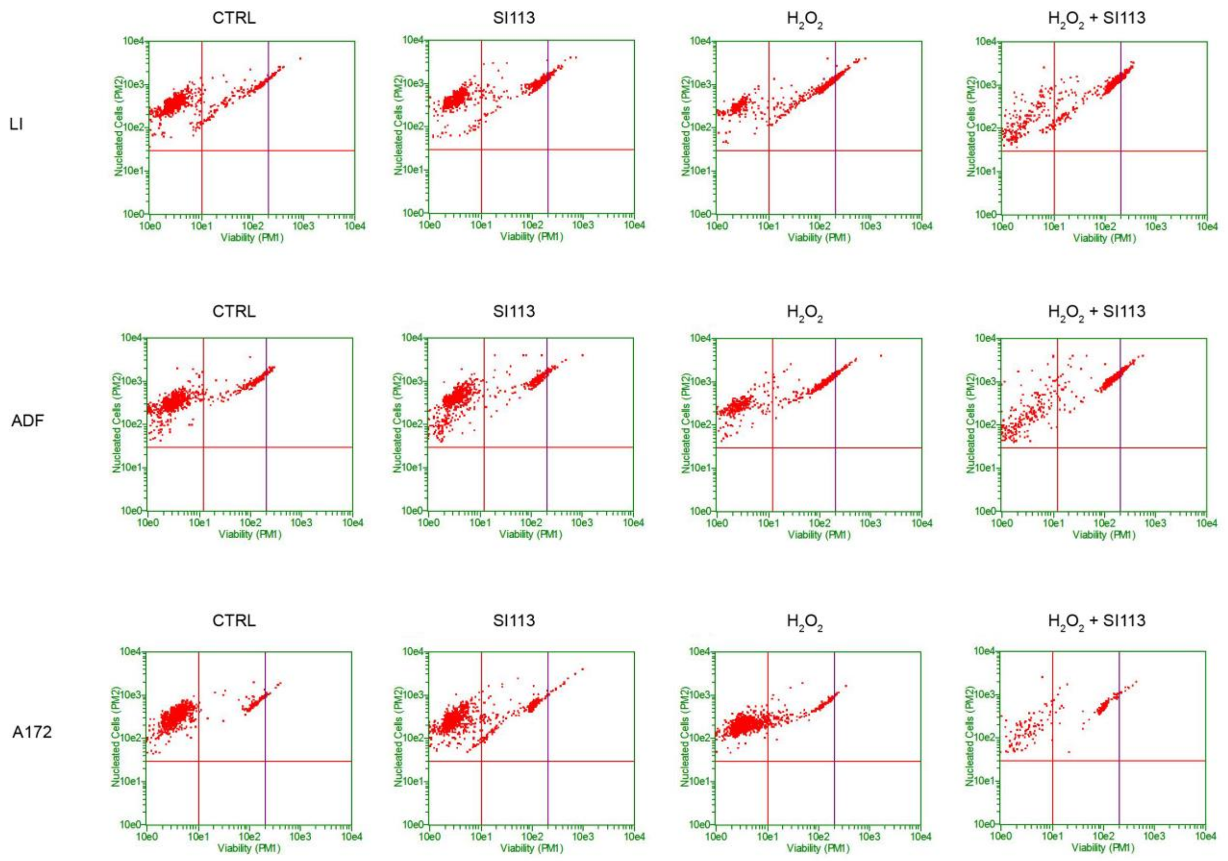

Supplementary Figure 7 Representative histograms of cells (LI, ADF and A172) treated or untreated with both H_2O_2 (250μM for 2h) and SI113 (12.5 μM) for 72 h and analyzed by Guava ViaCount flow cytometer

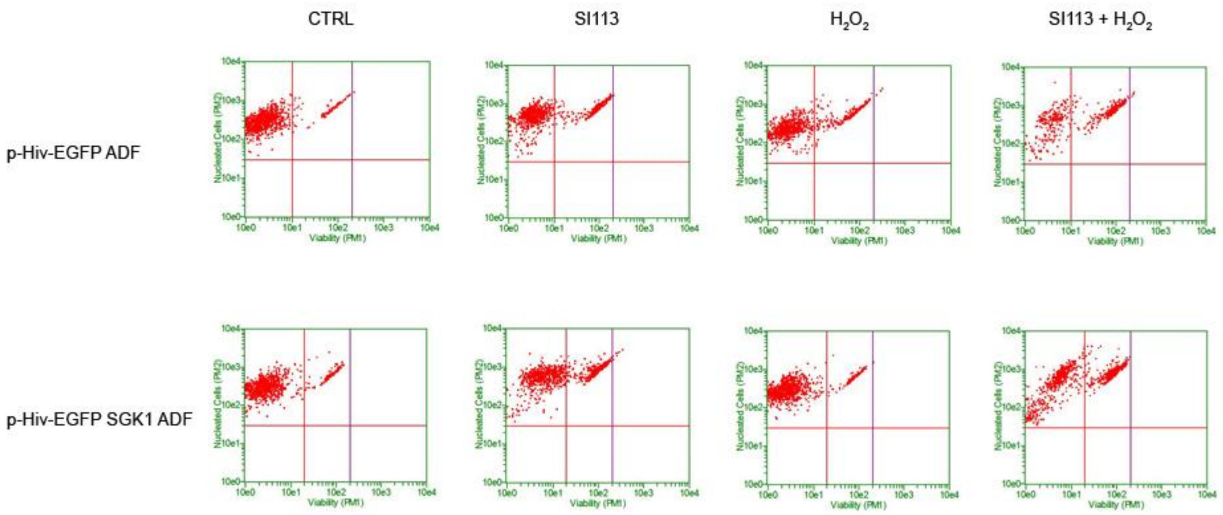

Supplementary Figure 8 Representative histograms of cells treated or untreated with both H_2O_2 (250μM for 2h) and SI113 (12.5 μM) for 72 h in both pHIV-EGFP ADF cells and pHIV-EGFP SGK1 ADF cells and analyzed by Guava ViaCount flow cytometer

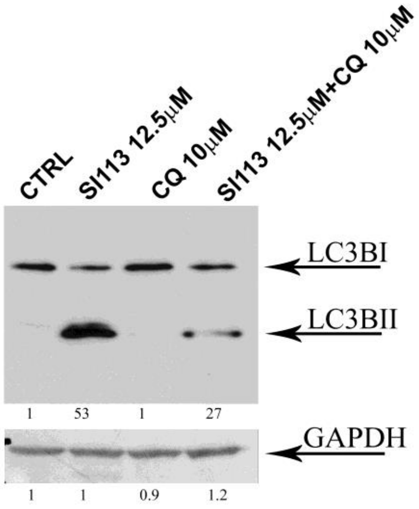

Supplementary Figure 9 WB for LC3B-I/II conversion in ADF cell line treated with 12.5μM SI113, CQ (10μM for 12h) or combined treatment with CQ used as pre-treatment. GAPDH was used as a loading control.

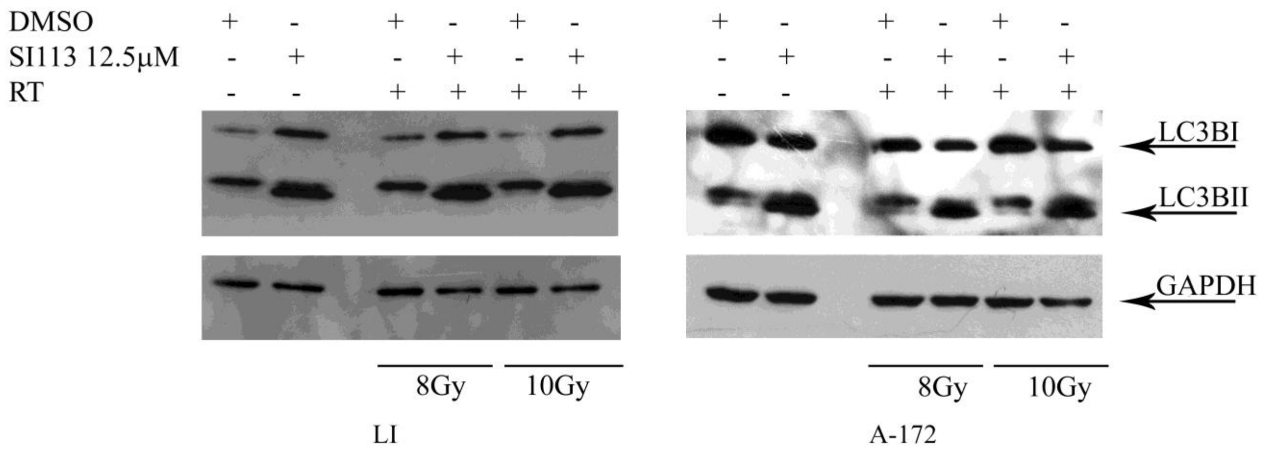

Supplementary Figure 10 WB for LC3B-I/II conversion in LI and A172 cell lines treated with 12.5μM SI113 for 72h. GAPDH was used as a loading control.
